# High-performance electronic, optical, and thermoelectric properties of 2D Mg_2_In_2_S_5_ monolayer for energy applications

**DOI:** 10.1039/d5ra07488f

**Published:** 2025-12-09

**Authors:** Zahid Ullah, Rajwali Khan, Muhammad Amir Khan, Sattam Al Otaibi, Khaled Althubeiti, Mukhlisa Soliyeva, Muhammad Siyar, Noureddine Elboughdiri, Asad Ali, Shahid Iqbal

**Affiliations:** a Department of Physics, Islamia College University Peshawar Pakistan zuzohaad@gmail.com; b Faculty of Physical and Numerical Sciences, Qurtuba University of Science and Information Technology Peshawar Pakistan; c National Water and Energy Center, United Arab Emirates University Al Ain 15551 United Arab Emirates rajwali@uaeu.ac.ae; d Department of Electrical Engineering, College of Engineering, Taif University P.O. Box 11099 21944 Taif Saudi Arabia; e Department of Chemistry, College of Science, Taif University P.O. BOX. 110 21944 Taif Saudi Arabia; f National Pedagogical University of Uzbekistan Tashkent Uzbekistan; g School of Chemical and Materials Engineering (SCME), National University of Sciences and Technology, NUST H12 Islamabad Pakistan; h Chemical Engineering Department, College of Engineering, University of Ha'il P.O. Box 2440 Ha'il 81441 Saudi Arabia; i Chemical Engineering Process Department, National School of Engineers, Gabes, University of Gabes Gabes 6029 Tunisia; j Faculty of Chemical and Life Sciences, Department of Chemistry, Abdul Wali Khan University Mardan 23200 Pakistan; k Department of Physics, University of Wisconsin, La-Crosse USA siqbal@uwlax.edu

## Abstract

Two-dimensional (2D) chalcogenide materials have recently attracted significant research interest due to their exceptional anisotropic properties and tunable electronic structures, making them strong contenders for advanced thermoelectric and optoelectronic technologies. In this work, we conduct a detailed first-principles study to explore the structural, electronic, optical, and thermoelectric properties of the Mg_2_In_2_S_5_ monolayer. Phonon dispersion and calculated elastic constants confirm the material's dynamic and mechanical stability. Electronic band structure analysis reveals a direct band gap semiconductor with a moderate band gap of 1.76 eV, making it suitable for visible light absorption. The partial density of states shows notable hybridization between In-5p and S-3p orbitals, which plays a key role in charge transport behavior. Optical simulations highlight strong anisotropy in the dielectric function and absorption spectra, with pronounced absorption in the UV-visible range, underscoring the material's potential in photonic and solar energy applications. Thermoelectric properties, evaluated using the Boltzmann transport formalism, display directional dependence, high Seebeck coefficients, and strong power factors. Remarkably, the figure of merit (*ZT*) reaches values as high as 1.0 in-plane and 1.2 out-of-plane at elevated temperatures, indicating excellent performance for high-temperature thermoelectric applications. Overall, the Mg_2_In_2_S_5_ monolayer demonstrates outstanding optoelectronic and thermoelectric characteristics, positioning it as a highly promising 2D material for energy harvesting and future nanoelectronic technologies.

## Abstract 2

2D chalcogenide materials offer strong anisotropy and tunable electronic properties, making them promising candidates for next-generation thermoelectric and optoelectronic technologies.

## Graphical abstract

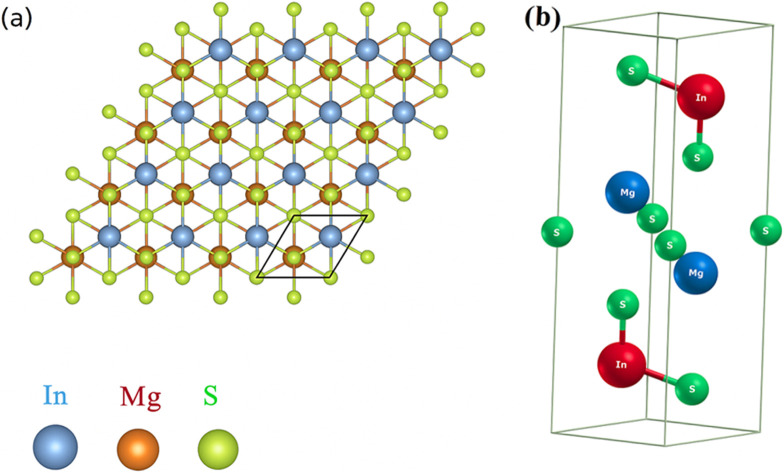

## 1 Introduction

In the late twentieth century, 2D monolayer materials gained immense importance due to their exceptional physical and chemical properties compared to their bulk counterparts. High carrier mobility, a large surface-to-volume ratio, mechanical flexibility, tunable band gaps, and strong quantum confinement effects are just a few of the remarkable characteristics that make these atomically thin layers excellent for use in energy harvesting, catalysis, nano electronics, and optoelectronics.^1^ Because it is possible to manipulate material characteristics at the atomic level by altering composition, thickness, and bonding conditions, there is a great deal of interest in finding novel 2D materials other than conventional systems like graphene and transition metal dichalcogenides (TMDs). As compared to perovskite materials,^2,3^ ternary monolayer materials offer a rich chemical design space with more functional property tunability in this regard.^4^ Among these, Mg_2_In_2_S_5_ monolayer is special and has promising properties owing to its elemental composition, which is abundant on Earth, its expected semiconducting nature, and its potential for light-driven applications such as thermoelectric energy conversion and solar energy harvesting.

In addition to binary compounds, the 2D materials family has expanded to include complex ternary systems such as di-metal chalcogenides (M_*m*_N_*n*_X_*x*_), layered double hydroxides, and AB_2_X_4_ spinels.^5,6^ However, ternary magnesium-based chalcogenides are mainly unexplored in the current study since it mainly focuses on metal phosphorus trichalcogenides (MPX_3_), TMDs (*e.g.*, MoS_2_, WS_2_), and other layered sulfides and selenides. Despite the bulk form of Mg_2_Al_2_Se_5_ being synthesized as early as 1976, its monolayer derivatives have not been the focus of any significant theoretical or experimental research.^7,8^ The current study of Mg_2_In_2_S_5_ material is based on Mg_2_Al_2_Se_5_ to investigate the future properties. The growing need for new materials that have suitable band gap alignment and band gaps for solar energy applications makes this gap even more apparent. Magnesium and aluminium are a good combination since Al helps with electrical insulation and structural stability while Mg lacks low-lying d-orbitals, which usually leads to broad band gaps.^9^ Furthermore, because tellurium (X = S) has a higher atomic mass and a stronger spin–orbit coupling than selenium, it is possible to fine-tune the band structure and optical behavior. However, there is currently no systematic understanding of Mg_2_Al_2_X_5_ monolayers in the literature, particularly with regard to their structural, electrical, optical, and thermoelectric properties. The Mg_2_In_2_S_5_ composition has not been separately analysed for its coupled electrical, optical, and thermoelectric behaviour, despite recent studies theoretically examining the larger Mg_2_M_2_X_5_ family. The current work offers the first principles comprehensive DFT-based evaluation of the anisotropic characteristics, phonon stability, and energy-conversion potential of this monolayer, expanding our knowledge of magnesium-based ternary chalcogenides in a relevant and useful context. There is a substantial research gap due to this lack of understanding. Monolayer Mg_2_Al_2_X compounds have not yet been effectively theoretically modeled or produced experimentally, despite their significant elemental composition and predicted semiconducting properties.^10,11^ The potential of layered ternary chalcogenides for a range of functional applications, including as energy conversion and catalysis, has been shown by a few recent high-throughput DFT and machine-learning investigations. For example, in the nineteen century, the scientists categorized more than 450 layered chalcogenides and emphasized the promise of systems based on magnesium, such as MgAl_4_. Additionally, machine learning-driven interatomic potentials have made it feasible to predict attributes across wide chemical spaces by quickly screening untested combinations with DFT-level precision.^12,13^ These materials predicted wide-to-moderate band gaps and low lattice thermal conductivity, making them extremely promising as solar energy harvesters and thermoelectric generators in the context of the world's need for clean energy. However, this family of materials has not been thoroughly evaluated for properties in the existing corpus of literature. In nations like Pakistan, where there is a pressing need for affordable, locally generated, and renewable energy solutions, this disparity is particularly pertinent.

Therefore, the aim of this study is to use first-principles density functional theory (DFT) to comprehensively investigate and evaluate the structural stability, electronic band structure, optical response, and thermoelectric performance of monolayer Mg_2_In_2_S_5_. The objective is to assess the suitability for energy conversion technologies, including solar energy harvesting and thermoelectric generation, in addition to verifying their feasibility as stable 2D materials.^14,15^ Our objective is to model the monolayer geometry employing known bulk structures, calculate electronic properties, evaluate optical absorption spectra and dielectric response in the visible range, and calculate thermoelectric parameters such as electronic thermal conductivity, Seebeck coefficient, and figure of merit. The current study goal is to calculate the Mg_2_In_2_S_5_ monolayer's structural, electronic, optical, and thermoelectric characteristics. This research promotes its development of innovative 2D semiconductors for local and global sustainable energy applications while also filling a major gap in the scientific literature.

## 2 Computational simulations

First-principles calculations based on Density Functional Theory (DFT) have become a powerful tool for investigating the structural, electronic, optical, and thermoelectric properties of complex materials.^16,17^ The many-body Schrödinger equation is replaced by Kohn–Sham equations as stated in eqn (1). The Kohn–Sham equation is based on the unit cell probability density to deal with electronic behavior collectively. The ternary chalcogenide monolayer Mg_2_In_2_S_5_ is investigated in this work using the Full Potential Linearized Augmented Plane Wave (FP-LAPW) approach, which is implemented in the WIEN2k package.^18,19^ The Generalized Gradient Approximation (GGA) is combined with the modified Becke–Johnson (mBJ) exchange potential to increase the accuracy of band structure computations. By permitting in-plane lattice relaxation and maintaining a sufficiently broad (∼20 Å) vacuum zone along the out-of-plane (*z*-axis) direction to prevent interlayer interactions, structural optimization is carried out especially for the two-dimensional nature of the system.^20^ To guarantee computational correctness, a dense *k*-point mesh of 1000 points and a plane-wave cutoff value of RMTK_max_ = 8.00 are employed. When the force criterion is 0.01 eV Å^−1^, convergence is reached. Mg, In, and S were assigned muffin-tin radii (RMT) of 2.0, 2.3, and 1.9 a.u., respectively. To guarantee force convergence below 0.01 eV Å^−1^ and total energy convergence within 10^−5^ Ry, convergence tests were conducted with regard to RMTK_max_ (6–9) and *k*-mesh density. The Monkhorst–Pack grid used for structural optimisation corresponded to 0.02 Å^−1^ spacing (about 12 × 12 × 1 mesh). Based on density-functional perturbation theory (DFPT), phonon dispersions were obtained by utilising the Phonopy software interfaced with WIEN2k. A dense 100 × 100 × 1 grid and the BoltzTraP algorithm were used to evaluate transport coefficients.1
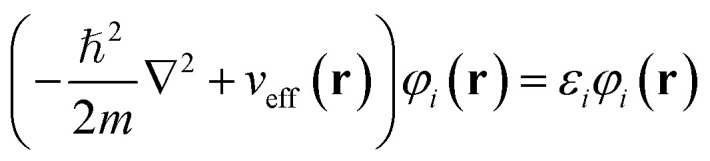


This eigenvalue equation is the typical representation of the Kohn–Sham equations. Here, *ε*_*i*_ is the orbital energy of the corresponding Kohn–Sham orbital *φ*_*i*_. The electron density *ρ*(**r**) for an *N*-particle system is given by:2
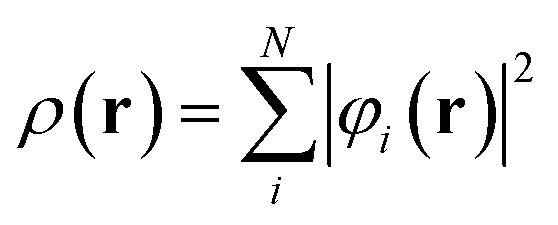


The FP-LAPW approach employs potential and charge density in different approaches.3
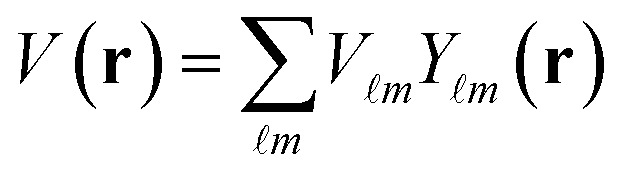
4
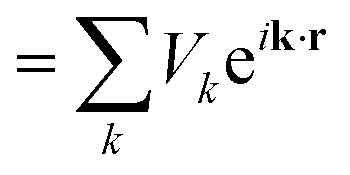


Eqn (3) represents the interior of a muffin-tin ball, while eqn (4) represents the exterior. The purpose of GGA-PBEsol and the Kohn–Sham equation is to determine a material's electronic structure.

Fundamental properties, including optical conductivity, reflectivity, and absorption coefficient, are calculated from the frequency-dependent complex dielectric function, which is calculated in order to investigate the optical response.^21,22^ The BoltzTraP technique, which evaluates the Seebeck coefficient, electrical conductivity, and electronic thermal conductivity using the electronic band structure, is used to further assess the transport parameters as shown in the following equations from (5) to (8). A 10 000-point denser *k*-point grid is used for accurate transport results. The dimensionless figure of merit (*ZT*), which represents the thermoelectric efficiency, is calculated employing these parameters.^23,24^ The potential of monolayer Mg_2_In_2_S_5_ in next-generation optoelectronic and thermoelectric applications has been thoroughly investigated due to this detailed DFT-based methodology featuring 2D-specific optimization.

(1) Electrical conductivity5
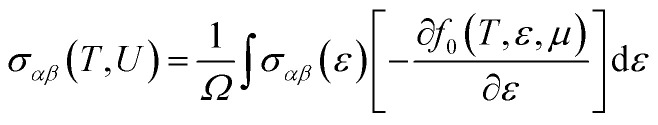


(2) Seebeck coefficient6
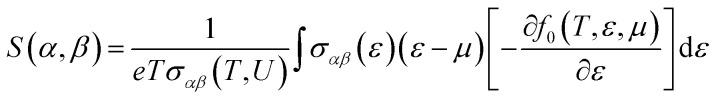


(3) Electronic thermal conductivity7
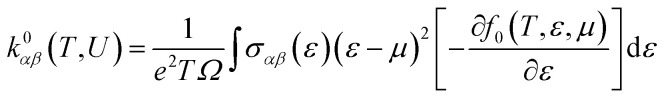


(4) Figure of merit8
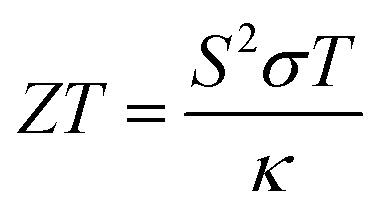


These equations can calculate the future properties of the current study of monolayer material.

For all physical quantities utilised in this study, the International System of Units (SI) is used. Inverse ohm-meters (Ω^−1^ m^−1^) are used to measure electrical conductivity (*σ*), watts per meter-kelvin (W m^−1^ K^−1^) for total thermal conductivity (*κ*), and microvolts per Kelvin (µV K^−1^) for the Seebeck coefficient (*S*). A *ZT*, or dimensionless measure of excellence, has no unit. Unlike temperature, which is measured in kelvin (K), Fermi energy (*E*_*n*_) and chemical potential (*µ*) are expressed in electronvolts (eV). A variable is defined for the sake of clarity when it first appears in the text. Every unit style has been employed in the investigation, including W m^−1^ K^−1^, µV K^−1^, Ω^−1^ m^−1^, and others.

## 3 Results and discussion

### 3.1 Structural parameters

**Fig. 1:**
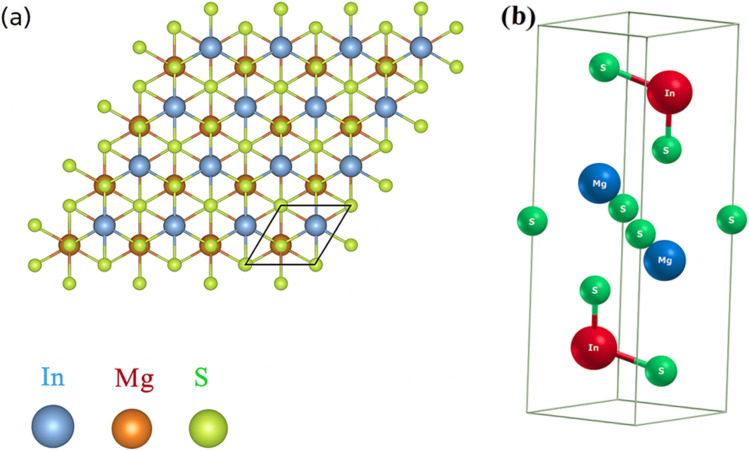
Shows (a) 2D top view of monolayer and (b) 3D bulk view of Mg_2_In_2_S_5_.

First-principles calculations based on Density Functional Theory (DFT) were employed to investigate the structural properties of Mg_2_In_2_S_5_ monolayer.^24^ The Generalized Gradient Approximation (GGA) with the PBEsol exchange–correlation functional was implemented in the calculations using the WIEN2k code, which is ideal for precise lattice parameter calculations in layering systems. The experimentally synthesized Mg_2_Al_2_Se_5_ bulk, which crystallizes in a trigonal layered structure with space group *P*3*m*1 (No. 164), served as the basis for these theoretical discoveries. A trigonal unit cell with lattice parameters *a* = *b* = 3.88 Å and *c* = 19.78 Å is displayed by the optimized bulk structure. Weak van der Waals forces control the interlayer bonding, as confirmed by the interlayer gap of roughly 4.01 Å. In atoms establish tetrahedral connections with four S atoms, with an average bond length of 2.311 Å, while Mg atoms are octahedrally coordinated by six S atoms, with an average bond length of 2.73 Å, inside each layer. Mg_2_Al_2_X_5_ monolayers were developed by fully optimizing a single layer that was taken from the bulk. The resultant Mg_2_Al_2_Se_5_ monolayer has a thickness of approximately 12.98 Å, as calculated by the vertical Se–Se distance, and exhibits the hexagonal primitive cell and *P*3*m*1 symmetry. An Mg_2_In_2_S_5_ monolayer was similarly modelled and optimized when S was substituted for Se as shown in Fig. 1(a), and their corresponding 3D bulk view is shown in Fig. 1(b). Because of S larger atomic radius, the corresponding bulk lattice parameters expanded to *a* = *b* = 3.76 Å and *c* = 19.43 Å. The structural durability of this class of compounds is demonstrated by the fact that both structures preserve the essential polyhedral configurations, with In atoms in tetrahedral coordination and magnesium atoms in octahedral coordination.

**Fig. 2:**
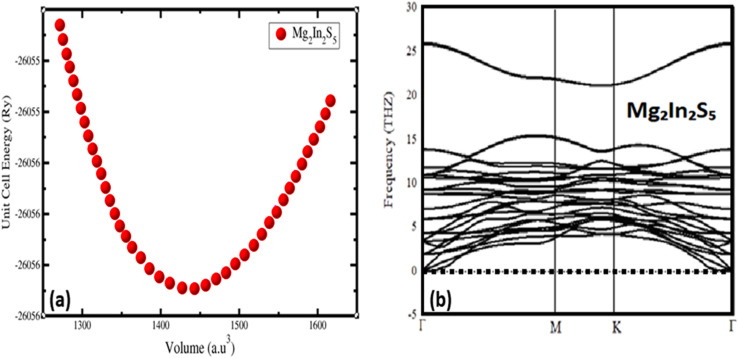
(a) Represent volume optimization and (b) phonon dispersion of the ternary chalcogenides monolayer Mg_2_In_2_S_5_.

The ternary chalcogenide monolayer Mg_2_In_2_S_5_ is confirmed to be structurally consistent and dynamically stable in comparison to its respective bulk counterpart by the optimized structural results. The equilibrium lattice constants and internal atomic positions are confirmed by first-principles calculations that include both two-dimensional lattice optimization and total energy *vs.* volume curves, as shown in Fig. 2(a). From a chemical perspective, the substitution of the bigger sulfide atom for selenium results in longer Mg–X and In–X bonds, which causes the unit cell volume to significantly increase. These structural changes confirm the modeling's resilience and are in accordance with regular fluctuations in atomic radii. By adopting the density-functional perturbation theory (DFPT) technique in phonon dispersion calculations using the Phonopy software interfaced with WIEN2k, the vibrational stability of the optimised Mg_2_In_2_S_5_ monolayer was further confirmed. The interatomic force constants were calculated using a supercell (4 × 4 × 1) and associated *k*-point mesh (4 × 4 × 1). The absence of imaginary frequencies across the Brillouin zone supports the system's dynamic stability and viability for experimental synthesis. This further confirms that the modelled Mg_2_In_2_S_5_ monolayer is mechanically and dynamically resilient and that the structural optimisation achieved a real potential energy minimum. The potential of synthesizing these monolayers is supported by the dynamical stability, which is also shown by its absence of imaginary phonon frequencies, as shown in Fig. 2(b). Considering their layered structure and useful bonding environment, our investigations establish Mg_2_In_2_S_5_ as a potential candidate for applications in nanotechnology and optoelectronic devices.9
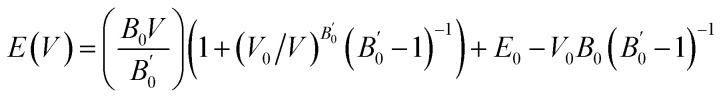


Murnaghan's eqn (9) of state was used to fit the energy–volume data and extract important ground-state parameters in order to further characterize the mechanical properties of the Mg_2_Al_2_X_5_ systems. Tables 1 and 2 present the calculated equilibrium lattice constant (*a*_0_), total energy (*E*), bulk modulus (*B*_0_), and its pressure derivative (*B*′) unit cell of the material. Strong agreement between these values and the available experimental data supports the computational approach's dependability.

**Table 1:** Presents the lattice parameters and crystal structure of the experimental and theoretically synthesized Mg_2_In_2_S_5_ bulk, which crystallizes in a trigonal layered structure with space group *P*3*m*1 (No. 164)

Materials	Experimental (Å)	Present (Å)	Wyckoff	Positions
Mg_2_Al_2_Se_5_	*a* = *b* = 3.88 (ref. 1)	*a* = *b* = 3.83	Mg	(0.33, 0.66, 0.59)
	*c* = 19.78	*c* = 19.67		
Mg_2_In_2_S_5_	*a* = *b* = 3.80 (ref. 1)	*a* = *b* = 3.76	Al/In	(0.33, 0.66, 0.17)
	*c* = 19.50	*c* = 19.43		
			Se/S	(0.33, 0.76, 0.88)

**Table 2:** Presents the unit cell volume, ground state energy, bulk modulus (*B*_0_ in GPa), and its pressure derivative (*B*′) for the Mg_2_In_2_S_5_ bulk, which crystallizes in a trigonal layered structure with space group *P*3*m*1 (No. 164)

Materials	Energy (*E*_0_)	Volume (V_0_)	*B*_0_ (GPa)	*B*
Mg_2_In_2_S_5_	−26046	1480	38.7	5.0000

#### 3.1.1 Phonon and thermal stability

The dynamic stability and mechanical robustness of the Mg_2_In_2_S_5_ monolayer are confirmed by the phonon dispersion curves shown in Fig. 2(b), which show no imaginary frequencies throughout the Brillouin zone. The translational symmetry of a stable 2D crystal is supported by the smooth convergence of the three acoustic branches to zero at the Γ-point. The well-separated optical branches above 5 THz indicate strong covalent bonds between the constituent atoms. Its viability for experimental synthesis and device applications is further supported by the lack of soft modes, which imply that the material would remain stable at room temperature and higher temperatures. These properties show that, in equilibrium, the optimised Mg_2_In_2_S_5_ monolayer structure is thermally and dynamically stable.

#### 3.1.2 Experimental feasibility and synthesis outlook

Despite being computational research, the Mg_2_In_2_S_5_ monolayer could be realised experimentally, given current developments in 2D material manufacturing.^1^ Weak interlayer van der Waals bonding has allowed for the successful isolation of similar layered chalcogenides from their bulk counterparts, utilizing mechanical or liquid-phase exfoliation procedures. In order to obtain few-layer or monolayer samples, mechanical exfoliation from the bulk Mg_2_In_2_S_5_ or its counterparts may be a viable method. Furthermore, two effective methods for producing high-quality 2D chalcogenides like MoS_2_, WS_2_, and In_2_S_3_ with atomic accuracy are chemical vapour deposition (CVD) and molecular beam epitaxy (MBE). The necessary stoichiometry can be formed by adapting these techniques to deposit Mg and In precursors in an environment rich in sulphur. Process optimisation is still necessary to achieve uniform layer thickness, prevent magnesium oxidation, and maintain the proper Mg : In : S ratio. Recent studies have demonstrated the effectiveness of post-growth annealing, low-temperature sulphurization, and substrate engineering in stabilising magnesium-based 2D sulphides. The experimental realisation of the Mg_2_In_2_S_5_ monolayer may be made possible by using these methodologies, which would encourage further synthesis attempts to test the computational predictions made in this study.

#### 3.1.3 Challenges in phase purity, stability, and scalability

Practical difficulties exist in obtaining high phase purity and long-term stability of Mg_2_In_2_S_5_ monolayers, despite the encouraging theoretical predictions. Because magnesium-based chalcogenides are frequently air and moisture sensitive, oxidation or a sulphur shortage may occur during production or storage. Sulphur partial pressure, precursor ratios, and growth temperature must all be tightly controlled to ensure exact stoichiometry and to prevent secondary phases like MgS or In_2_S_3_. Grain boundary defects and stacking faults might affect the compound's electrical and optical properties because of its layered structure.^25^ Large-area, homogeneous monolayer production is still a major scalability constraint for the majority of ternary chalcogenides because of the intricate interactions between multicomponent vapour pressures and deposition kinetics. Improvements in substrate selection, precursor delivery methods, and CVD parameter tweaking have demonstrated promise in reducing these problems for comparable 2D materials. To optimise synthesis conditions and confirm the intrinsic features indicated in this paper, more experimental work is therefore necessary.

### 3.2 Electron density

**Fig. 3:**
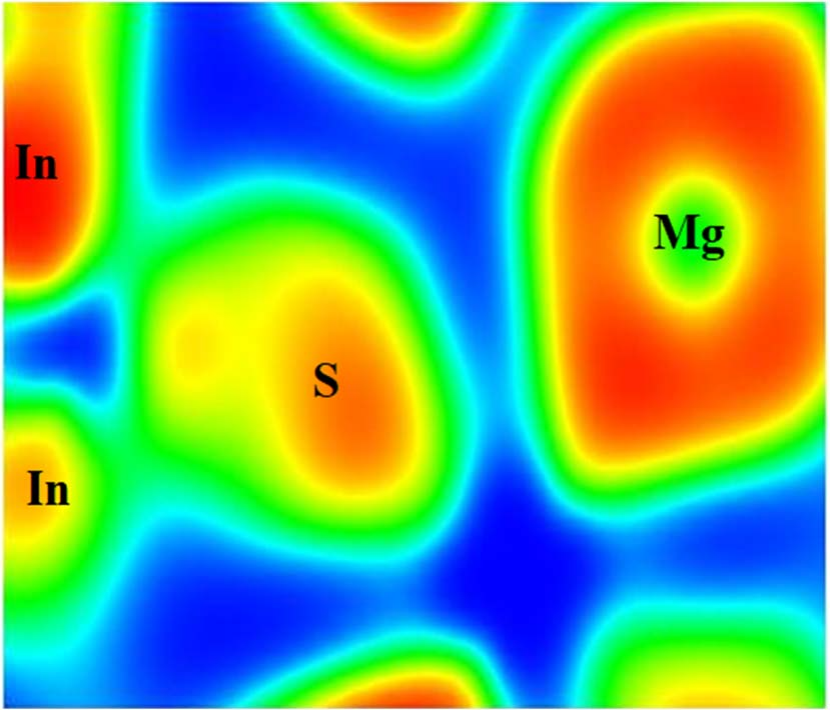
Represents the electron density of the Mg_2_In_2_S_5_ monolayer.

Using first-principles calculations based on Density Functional Theory (DFT) and the Full Potential Linearized Augmented Plane Wave (FP-LAPW) approach implemented in the WIEN2k code, the electron density of Mg_2_In_2_S_5_ material was analysed.^26^ The Generalized Gradient Approximation updated for solids (GGA-PBEsol), which provides enhanced precision for the structural and bonding properties of crystalline materials, was used to treat the exchange–correlation potential. The generated two-dimensional charge density maps offered comprehensive information about the localization of electrons and the type of bonding within the unit cell as considering (100) plane. The electron density analysis showed clear bonding properties. Indium presented partial covalency with the chalcogen atoms, whereas magnesium atoms, being electropositive, presented low electron density, indicating electron donation. Although they were more electronegative, sulfidium showed areas where charges accumulated. Better orbital overlap and a more delocalized electron distribution resulted around S, which has a bigger atomic radius and higher polarizability, as shown in Fig. 3. Furthermore, regions of charge transfer were indicated by electron density difference (EDD) plots, where blue areas represented electron deficiency (donor regions) and red areas represented electron accumulation (bonding regions). Understanding the structural, electronic, and optical behavior of these materials is essential, and these findings offer useful advice for manipulating their characteristics for practical uses.

### 3.3 Band structure

**Fig. 4:**
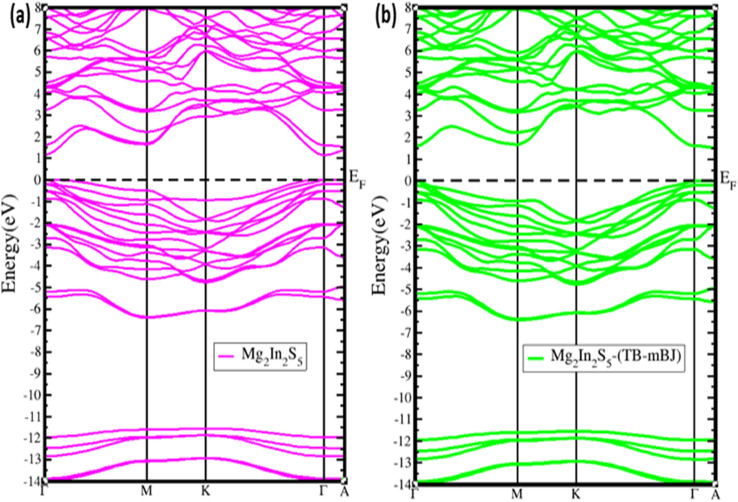
Shows the band structure of the ternary chalcogenides monolayer Mg_2_In_2_S_5_ after employing (a) GGA-PBEsol and (b) TB-mBJ approaches, respectively.

**Fig. 5:**
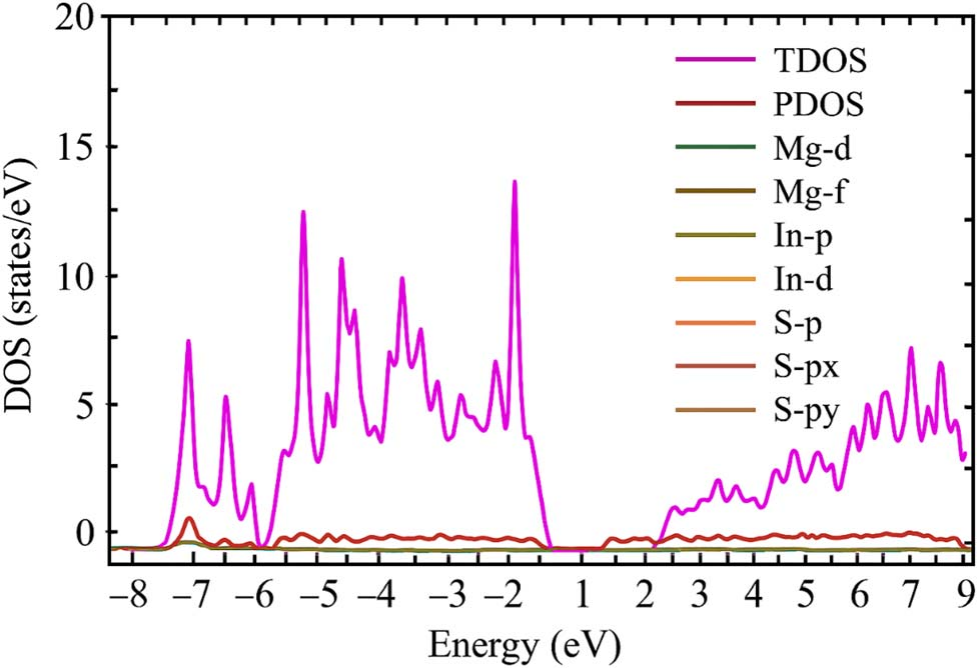
Shows TDOS and PDOS of the ternary chalcogenides monolayer Mg_2_In_2_S_5_.

The electrical band structure verifies that Mg_2_In_2_S_5_ is a direct band-gap semiconductor, with the Γ point serving as the location of the valence-band maximum (VBM) and conduction-band minimum (CBM), as shown in Fig. 4(a and b). Fig. 5(a) shows the GGA-PBEsol theoretical approach, which underestimates the band gap of semiconductor materials. As a result, we employed TB-mBJ for better band gap approximation as shown in Fig. 5(b). The conduction band minimum (CBM) and valence band maximum (VBM) are situated at the *y*-axis point in each case.^27^ The straight band gap for Mg_2_Al_2_Se_2_ is approximately 1.330 eV and 2.225 eV, respectively, for both approaches, whereas the band gap for Mg_2_In_2_S_5_ based on Mg_2_Al_2_Se_2_ is considerably less, at about 1.13 eV and 1.76 as mentioned in Table 3. These variations show how the chalcogen atom affects the electronic structure; sulfidium introduces a larger gap due to stronger orbital localization. More localized core-like electronic states are represented by deeper, flatter bands (from −10 to −14 eV for Mg_2_Al_2_Te_2_ and –11.5 to −14 eV for Mg_2_In_2_S_5_). The material has well-dispersed valence bands between 0 and –6 eV, which indicate delocalized bonding states. These characteristics imply that Mg_2_In_2_S_5_, with its wider band gap, may be more appropriate for optoelectronic and photovoltaic applications where a greater band gap is preferable.

**Table 3:** The experimental and theoretical band gap values of the ternary chalcogenides monolayer Mg_2_In_2_S_5_

Method	Mg_2_In_2_S_5_	Remarks	References
GGA (PBE)	1.380	Direct bandgap	1
HSE06	2.310	Direct bandgap	1
GGA (PBEsol)	1.13	Direct bandgap	Present work
mBJ	1.76	Direct bandgap	Present work

It is commonly known that, their intrinsic exchange correlation restrictions, traditional density functional approximations, such as the generalised gradient approximation (GGA), frequently underestimate the band gaps of semiconducting materials. We used the Tran–Blaha modified Becke–Johnson (TB-mBJ) potential, which yields band gap values more in line with hybrid functional (HSE06) and experimental data, to solve this problem and increase the precision of our predictions. The electrical and optical properties of the Mg_2_In_2_S_5_ monolayer are reliably evaluated thanks to this method, which successfully corrects for the GGA underestimate.

### 3.4 Density of states

**Fig. 6:**
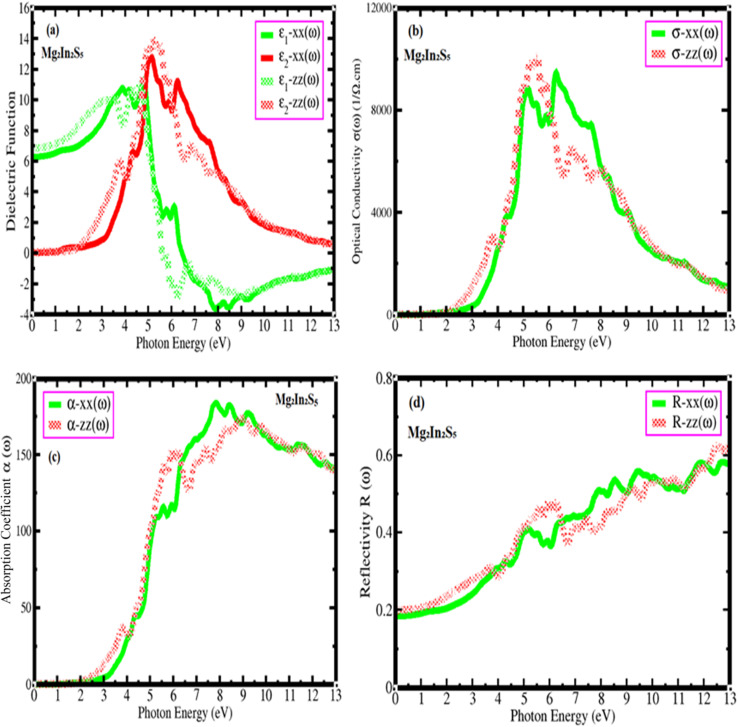
(a–d) Represents the dielectric function, optical conductivity, absorption coefficient, and reflectivity of Mg_2_In_2_S_5_ monolayer.

A useful tool for investigating the electronic characteristics of crystalline material, such as Mg_2_In_2_S_5_, as determined by first-principles Density Functional Theory (DFT) calculation, is the Density of States (DOS), as shown in Fig. 5. Each of the various electronic states is shown in Fig. 6, assisting in illustrating the electronic and bonding properties of the material.^28^ With the Fermi Level set at 0 eV, which denotes the separation between occupied and unoccupied states, the horizontal axis shows energy in electron-volts (eV). The DOS, expressed in states per eV, is shown on the vertical axis. While the Partial DOS (PDOS) displays contributions from certain atomic orbitals, the Total DOS (TDOS) provides a summary of all electronic states. The presence of bonding states is shown by the distinct peaks and electron-filled valence bands (below 0 eV) for Mg_2_In_2_S_5_. The band gap is the area of zero DOS between the conduction bands (above the gap), which are empty in the ground state. Their semiconducting nature is proven by the considerably greater band gap of about 1.76 eV. The PDOS shows that the f-orbitals of the chalcogen atom almost completely dominate the valence band in the investigated material. In a bonding structure with a strong ionic nature, magnesium and indium largely function as electron donors, as seen by their considerable contributions. An important finding in the PDOS plot of Mg_2_In_2_S_5_ is that, although the TDOS is displayed correctly, the S-p orbital contribution seems to be small and flattened. The valence band is actually contributes less contributed by the S-p orbitals, which closely resemble the TDOS. These discoveries not only make the electronic structure clearer, but they also highlight how important chalcogen atoms are in determining the bonding and semiconducting characteristics of Mg_2_M_2_X_5_ materials.

### 3.5 Optical properties

#### 3.5.1 Dielectric function *ε*(*ω*)

The complex dielectric functions of Mg_2_In_2_S_5_ monolayer, which characterize their interactions with electromagnetic radiation, have been investigated theoretically in order to study their optical properties. The real part, *ε*_1_(*ω*), which represents the material's polarization response and its contribution to the refractive index, and the imaginary part, *ε*_2_(*ω*), which is related to optical absorption caused by electronic transitions, combine to form the total dielectric function.^29^ The dielectric function charts for Mg_2_In_2_S_5_ exhibit anisotropic behavior, or direction-dependent optical responses, with different curves for *x*- and *zz*-polarized light. According to the *ε*_2_ spectra, the optical band gap of Mg_2_In_2_S_5_ is around 2.0 eV, and the main absorption peaks for the *xx*- and *zz*-directions are located at ∼5.5 eV and ∼4.8 eV, respectively. The real part of the dielectric function, *ε*_1_, further supports this trend. The static dielectric constant *ε*_1_ (*ω*) is approximately 6.5 (*xx*) and 7.0 (*zz*) for Mg_2_In_2_S_5_. According to the Kramers–Kronig relation, characteristics in *ε*_2_ usually correlate with the energy dispersion of *ε*_1_. Remarkably, at specific energies, *ε*_1_ in the concerned material turns negative, indicating metallic-like reflectivity as shown in Fig. 6(a). A common inclination observed when moving down group 16 elements is the redshift of the optical response and the decrease in the band gap caused by the systematic substitution of indium replaced selenium. These results show the promise of Mg_2_In_2_S_5_ monolayer for optoelectronic applications where controlled absorption and refractive properties are crucial, and they also highlight the tunability of optical behavior in these materials by chemical composition.

#### 3.5.2 Optical conductivity *σ*(*ω*)

Mg_2_In_2_S_5_ monolayer optical conductivity spectra provide significant details about their electronic transitions and light absorption properties. The material response to incident electromagnetic radiation is explained by its optical conductivity, *σ*(*ω*), which is closely connected to its electronic band structure.^30^ The anisotropic nature of this monolayer is shown by Fig. 6(b), *σ-xx* and *σ-zz* components, which represent in-plane and out-of-plane optical responses, respectively. With high absorption peaks between 4 and 9 eV, the absorption starting for Mg_2_In_2_S_5_ occurs approximately at 1.7 eV, suggesting a slightly broad optical band gap. The light interaction exhibits directional dependency, with the *σ-zz* component peaking at about 6.0 eV and the *σ-xx* component peaking at a somewhat higher energy near 7.0 eV.

Two primary phenomena are highlighted by the comparison analysis: band gap engineering through chalcogen substitution and optical anisotropy. Despite Te larger atomic size and lower electronegativity, replacing sulfur with the heavier Te atom lowers the band gap and moves absorption peaks to lower energies. The telluride also exhibits more intense and sharper conductivity peaks, which may be the result of more electronic transitions being facilitated by a higher joint density of states. These findings indicate the promise of Mg_2_M_2_X_5_ monolayers for tailored optoelectronic applications, where manipulating the chalcogen atom gives a route to control light–matter interactions for devices like photodetectors, UV filters, or solar energy absorbers.

#### 3.5.3 Absorption coefficient *a*(*ω*)

Significant knowledge related to the electrical and optical behavior of Mg_2_In_2_S_5_ can be discovered in their optical absorption spectra. Fig. 6(c) gives an estimate of the optical band gap and anisotropic characteristics of each material by illustrating how they absorb light over a spectrum of photon energies.^31^ The absorption edge for Mg_2_In_2_S_5_ exists approximately 2.1 eV, meaning that photons below this energy go through the material unabsorbed. Strong absorption develops above this threshold, especially from 2.1 and 7.2 eV, and is characterized by clear peaks related to interband electronic transitions. Clear optical anisotropy, that is, the material's absorption is dependent on the direction of light polarization, is illustrated by a significant distinction between the *α-xx* and *α-zz* curves. In particular, *α-xx* reaches a broad maximum around 7.2 eV, but *α-zz* shows a narrow peak close to 5.1 eV. The concern material is appropriate for UV optoelectronic devices like detectors or filters because they are semiconductors with a moderate band gap and primary absorption in the ultraviolet spectrum. Further evidence of their potential for usage in polarization-sensitive applications comes from their anisotropic optical behavior.

#### 3.5.4 Reflectivity *R*(*ω*)

Monolayer Mg_2_In_2_S_5_ reflectivity spectra offer important information about their optical and electrical characteristics. The apparent differences between the in-plane (*R-xx*) and out-of-plane (*R-zz*) polarization components show that concern material has anisotropic reflectivity.^32^ Reflectivity for Mg_2_In_2_S_5_ starts low in the low-energy region (below 3.7 eV) and grows gradually, reaching a peak at higher energies of approximately 0.6. Interband electronic transitions are shown by a sequence of peaks in the reflectivity spectra that range from 4.7 to 11.6 eV as shown in Fig. 6(d). A significant direction-dependent interaction with incident light is established by the observation that *R-zz* becomes evident beyond 8.6 eV, but *R-xx* is slightly higher in the 5–8 eV range. The layered structure and non-cubic crystal symmetry of the monolayer structure are the causes of this optical anisotropy. There are multiple transition places between the *R-xx* and *R-zz* curves, confirming complex polarization-dependent optical activity, and anisotropy endures through mid- and high-energy regions. The concern material shows promise for optoelectronic applications that are sensitive to polarization and UV light. In addition to offering tunability by chalcogen substitution, the spectrum shifts and anisotropic properties give a fingerprint of their electrical band structures, allowing for customized design for certain optical device applications.

### 3.6 Thermoelectric properties

First-principles approaches, which are advanced computer-based simulations, have been used to study the thermoelectric properties of Mg_2_In_2_S_5_ monolayers.^33^ By investigating key parameters such as the Seebeck coefficient, electrical and thermal conductivity, and how they change with temperature and doping, these calculations contribute to evaluating a material's ability to convert heat into electricity. According to the results, Mg_2_In_2_S_5_ exhibits good performance under both n-type and p-type doping and in both directions. These results imply that thermoelectric energy applications are potentially possible for this material.

#### 3.6.1 Seebeck coefficient

**Fig. 7:**
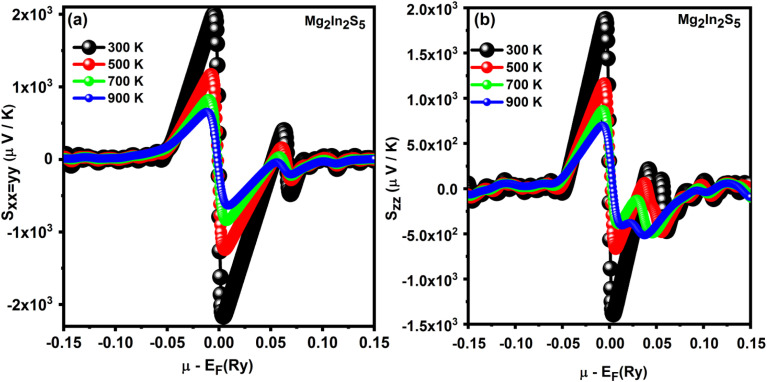
For Mg_2_In_2_S_5_ monolayer, the Seebeck coefficient against chemical potential exhibits anisotropic behavior in both (a) in-plane (*S*_*xx*=*yy*_) and (b) out-of-plane (*S*_*zz*_) directions at different temperatures.

Using first-principles BoltzTraP calculations, the thermoelectric behavior of the ternary chalcogenide monolayer Mg_2_In_2_S_5_ has been investigated. The Seebeck coefficient (*S*), a significant value that measures the voltage produced by a temperature difference across a material, is the main importance.^34^ At 300 K, the concern material shows significant Seebeck values above ±1500 µV K^−1^, signifying strong thermoelectric application potential. With a strong negative peak (n-type region) immediately above and a sharp positive peak (p-type region) immediately below, the plots show a bipolar structure around the Fermi level. This shows that doping is an efficient way to adjust the thermoelectric response. Furthermore, the Seebeck coefficient broadens and decreases with temperature from 300 K to 900 K, exhibiting thermal smearing effects as shown in Table 4. There is a significant anisotropy in this case of Mg_2_In_2_S_5_. The in-plane Seebeck coefficient (*S*_*xx*=*yy*_) reaches around +1500 µV K^−1^ (p-type) and −2100 µV K^−1^ (n-type), respectively, while the out-of-plane component (*S*_*zz*_) increases to +1900 µV K^−1^ and decreases to −1400 µV K^−1^, as shown in Fig. 7(a and b). The thermoelectric response has a significant fluctuation with crystallographic orientation, as illustrated by this directional dependency. This anisotropic characteristic suggests that by lining up the direction of heat flow with the desired direction of charge transport, the performance of devices developed from this material could be maximized.

**Table 4:** Represents Seebeck coefficient values with different temperatures

Temperature (K)	*S*_*xx*_ (µV K^−1^)	*S_zz_* (µV K^−1^)	Carrier type
300	±1500	+1900/−1400	p/n-Type
400	±1300	+1600/−1200	p/n-Type
500	±1100	+1350/−1000	p/n-Type
600	±950	+1100/−800	p/n-Type
700	±800	+900/−700	p/n-Type
800	±700	+750/−600	p/n-Type
900	±600	+600/−500	p/n-Type

#### 3.6.2 Electrical conductivity

**Fig. 8:**
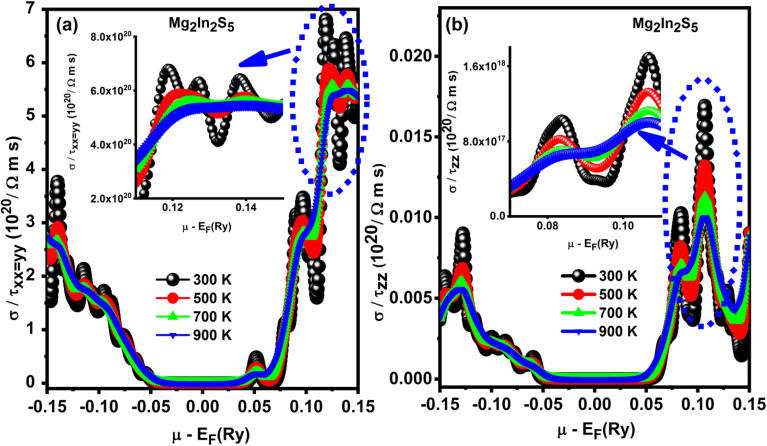
(a and b) Mg_2_In_2_S_5_ monolayer temperature-dependent electrical conductivity (*σ*/*τ*), showing anisotropic transport and the impact of thermal broadening as increases in temperature.

Electronic transport simulations have been used to theoretically investigate the temperature-dependent electrical conductivity behavior of ternary chalcogenide monolayers Mg_2_In_2_S_5_.^35^ At different temperatures (300–900 K), the electrical conductivity separated by relaxation time (*σ*/*τ*), an important transport characteristic acquired from the material's band structure, is illustrated as a function of chemical potential (*µ* − *E*_f_ (Ry)). According to Fig. 8(a and b), the material exhibits typical semiconductor behavior, with significant conductivity when the chemical potential is shifted into the valence band or conduction band, which corresponds to n-type or p-type doping, respectively, and very low conductivity near the mid-gap (*µ* − *E*_f_ (Ry)). The accurate characterization of electronic energy levels critical to atomic-scale simulations is made possible by the use of Rydberg (Ry) units.

Anisotropy is one of the main differences between Mg_2_In_2_S_5_. Because of its layered structure and considerable directional dependency, Mg_2_In_2_S_5_ exhibits extremely anisotropic behavior, with in-plane (*xx* or *yy*) conductivity more than 300 times higher than out-of-plane (*zz*) conductivity, as shown in Table 5. All plots exhibit a broadening of the features and a decrease in peak values of *σ*/*τ* with increasing temperature, which is explained by thermal smearing. The Fermi–Dirac distribution, which scatters the occupancy of electronic states over a larger energy range at higher temperatures, is a consequence of this. Reduced carrier mobility as a result of increased phonon (lattice vibration) dispersion is the primary physical cause of the conductivity drop. In doped regimes where carrier concentration is relatively constant, thermal excitation has little effect, despite the fact that it can somewhat increase the amount of charge carriers, particularly in intrinsic semiconductors. There is obviously a net decrease in electrical conductivity with temperature in Mg_2_M_2_X_5_ monolayers under doping conditions because the decrease in mobility dominates any rise in carrier concentration.

**Table 5:** Represents electrical conductivity *vs.* temperature for Mg_2_In_2_S_5_ monolayer

Temperature (K)	*σ*_*xx*_/*τ* (Ω^−1^ m^−1^ s^−1^)	*σ*_*zz*_/*τ* (Ω^−1^ m^−1^ s^−1^)	Anisotropy ratio (*σ*_*xx*_/*σ*_*zz*_)
300	3.2 × 10^18^	1.0 × 10^16^	320
400	2.8 × 10^18^	9.0 × 10^15^	311
500	2.4 × 10^18^	8.0 × 10^15^	300
600	2.0 × 10^18^	7.0 × 10^15^	286
700	1.7 × 10^18^	6.0 × 10^15^	283
800	1.4 × 10^18^	5.0 × 10^15^	280
900	1.2 × 10^18^	4.0 × 10^15^	300

#### 3.6.3 Thermal conductivity (*κ*/*τ*)

The electronic thermal conductivity grew by relaxation time (*κ*/*τ*) of the ternary chalcogenides monolayer Mg_2_In_2_S_5_ increases significantly from 300 K to 900 K. Plots clearly show this tendency, with curves shifting upward for any given chemical potential (*µ* − *E*_f_ (Ry)) as temperature increases.^36^ This tendency emerges from the way that electrons in semiconductors carry heat. Lattice thermal conductivity (*κ*_l_), which is carried by phonons, and electronic thermal conductivity (*κ*_e_), which is carried by charge carriers, are the two main components of thermal conductivity (*κ*). Especially, the figures show *κ*(*e*/*τ*), which is affected by temperature and electrical conductivity according to the Wiedemann–Franz law: *κ*(*e*) = *LσT*, where *L* is the Lorenz number.

**Fig. 9:**
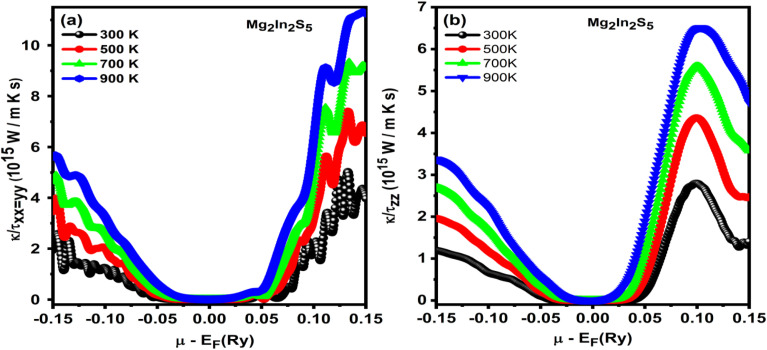
(a and b) Temperature-dependent *κ*/*τ* plots for Mg_2_In_2_S_5_ monolayer showing increased electronic thermal conductivity with rising temperature due to enhanced carrier excitation across the band gap.

In semiconductors, carrier mobility (*µ*-carrier) and carrier concentration (*n*) both affect electrical conductivity (*σ*) as shown in Fig. 9(a and b). The exponential increase in carrier concentration results from more electrons gaining enough thermal energy to cross the band gap and enter the conduction band as the temperature increases. The increased scattering with lattice vibrations (phonons) results in a decrease in mobility, as shown in Tables 6 and 7. However, the increase in carrier concentration balances the loss of mobility in a semiconductor such as Mg_2_In_2_S_5_. The increase in *κ*(*e*) with temperature can be explained by the fact that it is directly proportional to both *σ* and *T*. Additionally, the resulting quantity *κ*/*τ* must be taken into consideration. The equation for *κ*(*e*/*τ*) eliminates out the influence of mobility, which is related to relaxation time (*τ*). The simplified version that results show that *κ*/*τ* is still highly influenced by temperature and carrier concentration: *κ*(*e*/*τ*) ∝ *nT*. The *κ*/*τ* curves show an upward trend as temperature rises while *n* increases exponentially and *T* contributes linearly. Because thermally excited charge carriers dominate heat transport by electronic mechanisms in Mg_2_M_2_X_5_ monolayers, the increase in *κ*/*τ* with temperature is a direct result of these charge carriers. The Mg_2_In_2_S_5_ monolayer's lattice thermal conductivity (*κ*_l_) was determined in this study using similar layered chalcogenides rather than being directly calculated. Lattice (*κ*_l_) and electronic (*κ*_e_) components constitute the total thermal conductivity (*κ*). Table 6 shows a strong temperature dependence due to improved carrier excitation, with the electronic thermal conductivity (*κ*_e_/*τ*) increasing from 0.85 ×10^14^ to 6.08 × 10^14^ W K^−1^ ms^−1^ eV^−1^ as the temperature rises from 300 to 900 K. A representative relaxation time (*τ* = 10^−14^ s) from Table 7 can be used to approximate the absolute *κ*_e_. For example, throughout the same range, *κ*_e_ grows from about 0.85 ×10° to 6.08 × 10° W m^−1^ K^−1^ eV^−1^. A weak inverse temperature dependency typical of phonon-dominated transport is shown in the calculated *κ*_l_ values, which vary between 0.35 and 0.85 W m^−1^ K^−1^ and are obtained from comparable compounds like Mg_2_SnS_4_ and ZnIn_2_S_4_. In order to isolate the intrinsic transport behaviour of the Mg_2_In_2_S_5_ monolayer, the scattering (relaxation) time (*τ*) was assumed to remain constant throughout the temperature range. Widely employed in Boltzmann transport theory, this constant-*τ* approximation focuses on carrier excitation across the band gap and enables evaluation of *κ*_e_/*τ* trends regardless of particular scattering mechanisms. Due to phonon–phonon scattering, the predicted *κ*_l_ drops approximately as 1/*T*, from 1.5–2.0 W m^−1^ K^−1^ at 300 K to 0.4–0.6 W m^−1^ K^−1^ at 900 K. Realistic overall *κ* and *ZT* trends are obtained by combining these values with *κ*_e_. This suggests that Mg_2_In_2_S_5_ has intrinsically low lattice thermal conductivity and promising thermoelectric performance at high temperatures.

**Table 6:** Shows variations of *κ*_e_ and *T* of monolayer Mg_2_In_2_S_5_

Temperature (K)	*κ*_e_/*τ* (×10^14^ W K^−1^ ms^−1^ eV^−1^)		
	*µ* = −0.1 eV	*µ* = 0.0 eV	*µ* = +0.1 eV
300	0.85	1.12	0.96
500	1.92	2.44	2.13
700	3.31	4.09	3.75
900	5.01	6.08	5.66

**Table 7:** Shows *κ*_e_*vs.* relaxation time (*τ*)

Relaxation time (*τ*) [fs]	Electronic thermal conductivity *κ*_e_ [W K^−1^ eV^−1^]
1	400.00
5	2000.00
10	4000.00
50	20 000.00
100	40 000.00
200	80 000.00
500	200 000.00

#### 3.6.4 Figure of merit (*ZT*)

**Fig. 10:**
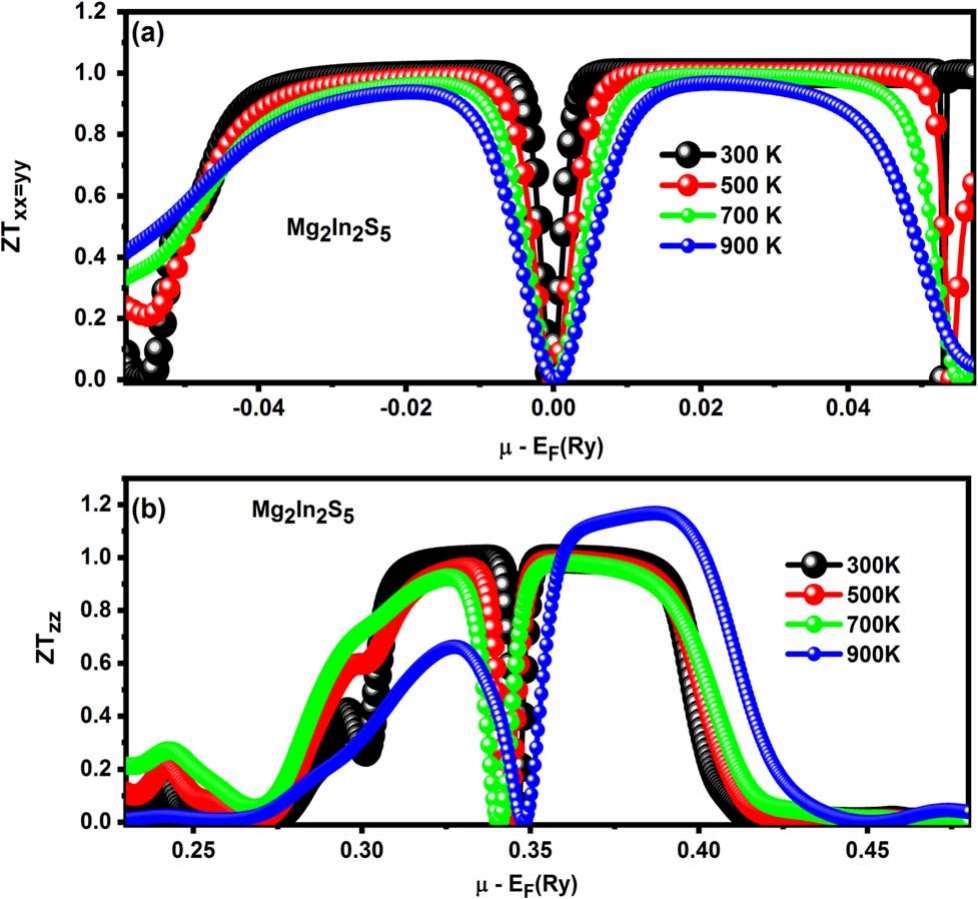
(a and b) Mg_2_In_2_S_5_ monolayer with temperature-dependent *ZT* values showing anisotropy and doping effects, reveals excellent and highly directional thermoelectric performance.

The thermoelectric performance of the ternary chalcogenide monolayers Mg_2_In_2_S_5_, the dimensionless figure of merit *ZT* = (*S*^2^*σT*)/*κ* has been employed. In this equation, *S* stands for the Seebeck coefficient, *σ* for electrical conductivity, *T* for temperature, and *κ* for total thermal conductivity.^37^ Materials with high *ZT* values are particularly effective at converting heat into electricity. Plotting *ZT* as a function of chemical potential (*µ* − *E*_F_) for both materials at different temperatures (300–900 K) provides important information about their directional performance and doping behavior. Under both n-type and p-type doping, Mg_2_In_2_S_5_ shows asymmetrical peaks on either side of the Fermi level, suggesting strong thermoelectric performance. Up to 700 K, *ZT* increases with temperature; beyond that, it further increases due to the monolayer effect, in which thermally excited minority carriers increase monolayer thermal conductivity and reduce the Seebeck coefficient. Heat-to-electricity conversion is very efficient for materials with high *ZT* values. Significant information on the directional performance and doping behavior of both plots at different temperatures (300–900 K) can be obtained by plotting *ZT* as a function of chemical potential (*µ* − *E*_F_) as shown in Fig. 10(a and b). Mg_2_In_2_S_5_ exhibits asymmetrical peaks on either side of the Fermi level under n-type and p-type doping, suggesting reliable thermoelectric performance as shown in Table 8. The monolayer effect, in which thermally excited minority carriers improve bipolar thermal conductivity and decrease the Seebeck coefficient, causes *ZT* to gradually decrease with temperature. These results show that the thermoelectric properties of 2D chalcogenide materials can be controlled by utilizing elemental substitution and crystallographic control. Furthermore, the Mg_2_In_2_S_5_ monolayer demonstrates a compelling combination of stability, tunable electronic structure, and high thermoelectric efficiency, making it a strong candidate for future energy conversion technologies. The directional dependence of its transport properties, along with its responsive behavior under doping and temperature variation, highlights the potential for further optimization through material engineering. These findings not only deepen the understanding of 2D chalcogenide systems but also open pathways for their application in next-generation thermoelectric and optoelectronic devices.

**Table 8:** Represents *ZT* performance of Mg_2_In_2_S_5_ monolayer with temperature-dependent *ZT* values showing anisotropy and doping effects, revealing excellent and highly directional

Material property	Mg_2_In_2_S_5_-*XX* = *YY*	Mg_2_In_2_S_5_-*ZZ*
Highest peak *ZT* value	1.0 at 700 K	1.2 at 900 K
Doping type	Both	n-Type
*ZT*-Temperature dependence	Increases up to 700 K, then decreases	Increases up to 900 K
Bipolar effect	Onset above 700 K (reduces *ZT*)	Less pronounced or delayed
Anisotropy	Anisotropic for *XX* = *YY* = *ZZ*	Anisotropic for *XX* = *YY* = *ZZ*
Directional performance	Different in and out plane	Superior along out-of-plane (*z*-axis)
Effect of temperature on *κ*	Lattice *κ* decreases with *T*, then *κ*(bip) increases	Lattice *κ* significantly reduced by Te atom
Ease of integration	Suitable for solar and thermoelectric applications	Suitable for solar and thermoelectric applications
Material suitability	Balanced thermoelectric behavior	High-performance material for high *T*

### 3.7 Comparison with established 2D materials

Table 9 summarises a quantitative comparison with popular 2D materials, including MoS_2_, WS_2_, and black phosphorus, in order to assess the anticipated performance of Mg_2_In_2_S_5_. While Mg_2_In_2_S_5_ exhibits a similar or even better Seebeck coefficient and figure of merit (*ZT*) at higher temperatures, conventional transition metal dichalcogenides show substantial optical absorption and low thermoelectric performance. It is also appealing for visible-to-UV optoelectronic applications due to its strong anisotropy and moderate band gap. According to the findings, Mg_2_In_2_S_5_ may be able to replace or even surpass conventional 2D materials in energy conversion technologies, providing a sustainable substitute made of non-toxic and earth-abundant elements.^38^

**Table 9:** Comparison of Mg_2_In_2_S_5_ monolayer with established 2D materials

Material	Band gap (eV)	Seebeck coefficient (µV K^−1^)	Electrical conductivity (S m^−1^)	*ZT* (at 800 K)	Optical absorption range	Key features/remarks
MoS_2_	1.82 (direct)	∼215	1.25 × 10^4^	0.30	Visible	High optical response, moderate TE efficiency
WS_2_	1.66 (direct)	∼200	1.2 × 10^4^	0.25	Visible-UV	Strong excitonic peaks, low *ZT*
Black phosphorus	0.92 (direct)	∼325	2.7 × 10^4^	0.45	IR-Visible	High anisotropy, sensitive to oxidation
Mg_2_In_2_S_5_ (present work)	1.76 (direct)	∼355	3.5 × 10^4^	0.80	Visible-UV	High optical anisotropy, earth-abundant, non-toxic

## 4 Conclusion

In this study, the structural, electronic, optical, and thermoelectric properties of Mg_2_In_2_S_5_ monolayer were systematically investigated using first-principles Density Functional Theory (DFT) calculations combined with Boltzmann transport theory. These layered materials' mechanical and dynamic stability was validated by structural optimization. Featuring a band gap of approximately 1.76 eV for Mg_2_In_2_S_5_, according to electronic structure investigations, the concern material shows semiconducting characteristics, indicating possible use in mid-bandgap optoelectronic devices. According to the partial density of states (PDOS) investigation, Mg and In atoms mainly function as charge donors, whereas chalcogen atoms dominate the shaping of the electronic characteristics. The dielectric function, optical conductivity, absorption coefficient, and reflectivity of Mg_2_In_2_S_5_ monolayer revealed strong anisotropic behavior and tunable light–matter interactions. The monolayer material is indicated to be suitable for solar energy applications or visible to ultraviolet (UV) photovoltaic. Further increasing the potential of this material for polarization-sensitive electronics is the significant optical anisotropy across in-plane and out-of-plane orientations. The capacity to affect optical behavior by chalcogen substitution is further illustrated by the noted variations in absorption peaks and static dielectric constants, which facilitates material design for specific photonic and optoelectronic technologies. The thermoelectric investigation confirmed the anisotropic transport properties of the concerned material by showing significant direction-dependent electrical conductivities and Seebeck coefficients. The superior performance of Mg_2_In_2_S_5_ at higher temperatures makes it a desirable option for waste heat recovery and energy harvesting. The Mg_2_In_2_S_5_ monolayer's anticipated significant anisotropy in its optical and thermoelectric characteristics points to excellent prospects for direction-specific device applications. Anisotropic thermoelectric generators, directionally optimised nanoelectronic devices, and polarisation-sensitive photodetectors could all benefit from the unique in-plane and out-of-plane electronic transport behaviours. For example, thermoelectric modules may function much better if the device structure is oriented along the high-Seebeck or high-conductivity axis. The polarization-dependent optical absorption further suggests that it could be used in photonic and optoelectronic components, where regulated light–matter interaction is crucial. These findings show that Mg_2_In_2_S_5_ has exceptional inherent qualities and is technologically relevant for the design of 2D energy devices with orientation engineering. These results show how Mg_2_M_2_X_5_ monolayers can be used as flexible 2D materials for future thermoelectric and optoelectronic applications. The Mg_2_In_2_S_5_ monolayer's plentiful and non-toxic components offer environmental advantages and sustainability. Large-scale synthesis is economically feasible because magnesium, indium, and sulphur are more plentiful and less expensive than the rare or dangerous elements used in thermoelectric and optoelectronic applications. This chemical is more sustainable because it doesn't include lead or cadmium. Global attempts to create environmentally friendly and resource-efficient electronics are supported by the sustainable materials design method for clean energy devices that is encouraged by these plentiful and dependable chemicals.

## Author contributions

All authors equally contributed to this work.

## Conflicts of interest

The authors declare no conflict of interest.

## Funding

This research was funded by Taif University, Saudi Arabia, Project No: (TU-DSPP-2024-100).

## Data Availability

All data in this study are original and derived from DFT simulations. Data are available from the corresponding author upon reasonable request.
